# Fully Green Particles Loaded with Essential Oils as Phytobiotics: A Review on Preparation and Application in Animal Feed

**DOI:** 10.3390/antibiotics14080803

**Published:** 2025-08-06

**Authors:** Maria Sokol, Ivan Gulayev, Margarita Chirkina, Maksim Klimenko, Olga Kamaeva, Nikita Yabbarov, Mariia Mollaeva, Elena Nikolskaya

**Affiliations:** N.M. Emanuel Institute of Biochemical Physics RAS, Moscow 119334, Russia; gulyaev@sky.chph.ras.ru (I.G.); chir.marg@sky.chph.ras.ru (M.C.); klimenko@sky.chph.ras.ru (M.K.); olekamaeva@gmail.com (O.K.); yabbarov_ng@sky.chph.ras.ru (N.Y.); mollaeva-mr@sky.chph.ras.ru (M.M.)

**Keywords:** essential oils, encapsulation, bio-based material, animal nutrition, phytobiotics

## Abstract

The modern livestock industry incorporates widely used antibiotic growth promoters into animal feed at sub-therapeutic levels to enhance growth performance and feed efficiency. However, this practice contributes to the emergence of antibiotic-resistant pathogens in livestock, which may be transmitted to humans through the food chain, thereby diminishing the efficacy of antibiotics in treating bacterial infections. Current research explores the potential of essential oils from derived medicinal plants as alternative phytobiotics. This review examines modern encapsulation strategies that incorporate essential oils into natural-origin matrices to improve their stability and control their release both in vitro and in vivo. We discuss a range of encapsulation approaches utilizing polysaccharides, gums, proteins, and lipid-based carriers. This review highlights the increasing demand for antibiotic alternatives in animal nutrition driven by regulatory restrictions, and the potential benefits of essential oils in enhancing feed palatability and stabilizing the intestinal microbiome in monogastric animals and ruminants. Additionally, we address the economic viability and encapsulation efficiency of different matrix formulations.

## 1. Introduction

Antibiotic resistance is a major problem in agriculture due to the widespread use of antibiotics in livestock [1]. Farmers often add antibiotic growth promoters (AGPs) to animal feed at low, sub-therapeutic doses to boost growth and improve feed efficiency. AGPs suppress bacterial growth in the gut, but their continued application contributes to the emergence of antibiotic-resistant strains, which pose a serious threat to food safety and public health.

Resistant bacteria can be transmitted from animals to humans in several ways. One of the most common pathways is through food. For instance, raw milk may carry antimicrobial resistance genes. When people consume unprocessed animal products, resistant strains can interfere with the human microbiota [2]. These strains can also contaminate soil and groundwater. Farm workers may become infected through direct contact or contaminated surfaces and further transmit these bacteria [3]. As a result, many countries banned or restricted AGP application in animal feed [4]. The European Union banned antibiotics as growth promoters in 2006 [5]. China applied a similar ban in 2020 [6]. These regulatory restrictions increased the demand for safe and effective alternatives to antibiotics in animal nutrition. Rising consumer demand for antibiotic-free, sustainably produced animal products is driven by increased awareness of human health, food safety, animal welfare, and environmental concerns. This trend is accelerating research into antibiotic alternatives, such as phytobiotics, as producers and retailers respond to evolving consumer preferences and regulatory pressures.

Essential oils (EOs) have emerged as a promising alternative to antibiotics in animal nutrition. These multicomponent mixtures, extracted from aromatic plants by distillation or solvent application, fall under the category of phytobiotic feed additives. Their antimicrobial and growth-promoting effects attracted growing attention [7]. EOs improve feed consumption, stimulate digestive enzyme secretion, enhance gut motility, and possess antiviral, antiparasitic, antifungal, immunomodulatory, antioxidant, and anti-inflammatory properties [8]. Encapsulating EOs can enhance their stability, protect them from degradation, allow for controlled release, and minimize the loss of volatile components [9].

Current research favors the application of natural, eco-friendly encapsulation matrices. These matrices are generally food-grade, biodegradable, and capable of forming a barrier between the EOs and the external environment [10]. Application of natural matrices like lipids, proteins, and polysaccharides reflects the industry’s shift toward greener solutions [11]. This approach also aligns with the “clean, green, and ethical” concept in farm management, where smart nutrition plays a central role [12].

Encapsulated EOs are approved for animal feed use in major markets, including the EU, the US, and China, with regulatory specifics varying by region and EO type. In the EU, EFSA has assessed the safety and efficacy of certain EOs (e.g., clove bud and leaf oil) as sensory feed additives across species. These oils are considered safe at specified concentrations and effective without further efficacy proof, given their flavoring role similar to food use [13]. The US feed encapsulation market is rapidly expanding, driven by demand for nutrient targeting and feed efficiency. Microencapsulation holds a 37.6% share in 2025, commonly applied to EOs, enzymes, and probiotics to protect actives, control release, and mask undesirable flavors [14]. The US leads this market due to advanced livestock systems and supportive regulation, though detailed lists of approved encapsulated EOs products remain proprietary. In China, detailed regulatory data on EO-encapsulated feed additives are limited. However, China represents a major East Asian market segment, motivated by sustainable and efficient livestock nutrition. The use of encapsulated EOs additives is nascent but projected to grow alongside regulatory developments and market demand [14].

In this review, we focus on current methods for encapsulating EOs to improve their stability and efficacy, along with their application in animal nutrition. We discuss the scalability of each encapsulation technique and summarize the key economic and physicochemical characteristics of various matrix types. Additionally, we highlight emerging trends in the production of EO-loaded particles. Overall, this review outlines recent advances in EOs encapsulation and their potential to enhance performance indicators in animal production.

## 2. Essential Oils as Phytobiotics

### 2.1. Origin, Classification, and Structure

Essential oils are added to animal diets for three main reasons. Firstly, they are increasingly acknowledged as efficacious alternatives to conventional antibiotics, offering a crucial advantage by mitigating the development and spread of bacterial resistance—a major challenge in contemporary animal husbandry. Secondly, beyond their antimicrobial efficacy, essential oils enhance digestive processes and promote improved growth performance, thereby facilitating optimized feed efficiency and healthier animal development. Thirdly, their inclusion aims to augment inherent qualities of animal-derived products, potentially improving parameters such as product quality, shelf-life, and sensory characteristics, thus providing added value along the food production chain [15].

**Figure 1:**
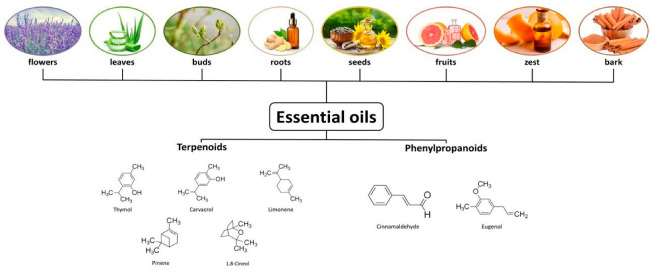
Origin, classification, and structure of antibacterial components of essential oils. EOs are volatile constituents obtained from aromatic plant material, including leaves, flowers, roots, bark, seeds, fruits, buds, and zest [20]. The most common classes of chemical compounds found in EOs are terpenoids and phenylpropanoids [21].

The wide variety of bioactive compounds in EOs makes it difficult to determine a specific mechanism responsible for their antibacterial activity. Gauthier identifies several compounds that exhibit the greatest antibacterial activity (Figure 1). Among them are monoterpenes (thymol, carvacrol, limonene, pinene, 1,8-cineol) and phenolpropanoids (eugenol, cinnamaldehyde) [16]. The main components of the various essential oils are well-documented in the literature [17]. For example, thymol is the main component of the aerial parts of *Thymus vulgaris* and the leaves of *Guazuma ulmifolia* [18], while cinnamaldehyde and eugenol are abundant in flower buds of *Eugenia caryophyllus* [19].

### 2.2. Mechanism of Action

**Figure 2:**
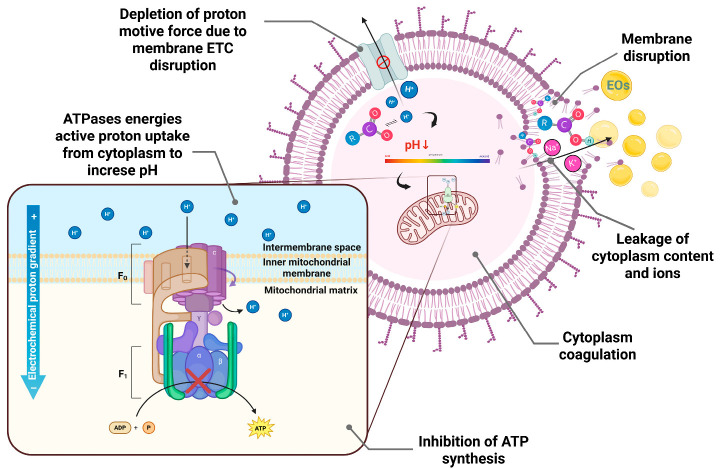
Mechanism of antibacterial synergistic action of essential oils and organic acids. Essential oils disrupt the bacterial cell membrane. This damage diminishes the proton-motive force and lowers intracellular ATP levels, leading to a loss of energy metabolism and, eventually, cell death [23]. Organic acids complement this effect by compromising the bacterial cell wall integrity.

The antibacterial effect of EOs largely derives from their hydrophobic nature, which gives them a strong affinity for bacterial cell membrane lipids [22]. Gram-negative bacteria are less sensitive to EOs compared to Gram-positive bacteria due to their more complex outer membrane structure [16]. However, monoterpenes such as thymol and carvacrol demonstrated efficacy against Gram-negative strains as well [22]. Essential oils destabilize the cell membrane, leading to its rupture, leakage of cellular contents and ions, and coagulation of the cytoplasm. Increased membrane permeability reduces the proton-motive force and the amount of intracellular adenosine triphosphate (ATP) molecules by decreasing ATP synthesis. Ultimately, the cell loses its ability to maintain energy metabolism, which can result in cell death (Figure 2) [23,24,25].

Organic acids can enhance the antibacterial activity of EOs. Undissociated organic acid disrupts the integrity of the bacterial cell wall. In the neutral environment of the cytoplasm, the acid dissociates into protons and anions. The latter are toxic to the cell and inhibit metabolic reactions, including those involved in ATP formation and the synthesis of essential enzymes. The increase in cytoplasmic protons lowers the internal pH, which inhibits essential bacterial enzymes. At the same time, the cell must use ATPase activity to transport the excess protons, further taxing its energy metabolism (Figure 2). The resulting anions interfere with key metabolic processes, including RNA and DNA synthesis. These disruptions inhibit bacterial growth and reproduction [16,26,27].

### 2.3. Impact on Pathogenic Microflora

EO concentration plays a critical role in determining antibacterial efficacy. Ouwehand and colleagues found that the Gram-positive *Clostridium perfringens* was especially sensitive to cinnamaldehyde and citral. Rosemary oil at 5 mg/L exhibited similar activity against *C. perfringens* as the commercial antibiotic avilamycin. In the case of Gram-negative *Salmonella enterica*, compounds such as carvacrol, cinnamaldehyde, citral, and thymol showed strong antibacterial effects at high concentrations (500 mg/L). Notably, thymol and carvacrol inhibited *Escherichia coli* strains even at low concentrations (5 mg/L) [28].

### 2.4. Impact on Probiotic Microflora

The effect of EOs on probiotic microflora remains uncertain, as some studies report both inhibitory and stimulatory outcomes. *Bifidobacterium longum* and *B. breve* showed resistance to thymol, eugenol, and carvacrol at 300 mg/L. Among *Lactobacillus* species, *L. plantarum* tolerated these EOs under aerobic conditions at the same concentration, while *L. acidophilus* was more sensitive [29]. *L. reuteri* displayed sensitivity to thymol and carvacrol, but only at higher doses (500 mg/L) in an aerobic, gut-like environment. Interestingly, compounds such as carvacrol, cinnamaldehyde, thymol, limonene, oregano oil, and thyme oil stimulated the growth of *L. fermentum* [28]. In another study, cumin powder at 0.5–2.0% (*w*/*w*) promoted the growth and acid production of *L. plantarum* in liquid medium, whereas cumin EO inhibited growth at higher concentrations (300–600 ppm) [30]. These findings suggest that the effects of EOs on probiotic bacteria are concentration-dependent. However, the specific mechanisms underlying these selective actions remain unclear.

Selection of encapsulation matrices and release strategies is critical to optimize encapsulation for preserving beneficial *Lactobacillus* spp. while inhibiting pathogens.

Complex coacervation using oppositely charged polyelectrolytes (e.g., gum arabic, chitosan, alginate, proteins) forms robust encapsulation networks that improve *Lactobacillus* viability, demonstrated with strains, including *L. plantarum*, *L. casei*, and *L. rhamnosus* GG [31]. Co-encapsulation of probiotics with bioactives like omega-3 fatty acids in hydrogel beads or microcapsules further enhances functional delivery [31].

Microencapsulation protects volatile and labile EOs from degradation, enabling targeted release within the gastrointestinal tract, prolonging residence time, and maximizing interaction with intestinal microbiota. Controlled release systems enable sustained delivery of antimicrobial agents [32].

## 3. The Need and Benefits of EOs’ Encapsulation

The free form of EOs has several drawbacks, primarily due to the presence of volatile organic compounds (VOCs). The most common VOCs with antibacterial properties are α-pinene, limonene, linalool, alpha-phellandrene, beta-myrcene, and camphene [33,34,35,36,37,38,39]. VOC evaporation reduces the potential positive effects of consumed EOs. On the other hand, prolonged exposure to VOC emissions can lead to respiratory illnesses in farm and factory workers. Many EOs contain compounds like limonene, acetone, ethanol, acetaldehyde, and methanol, known for their potential toxicity [38].

EOs are also sensitive to environmental factors such as ultraviolet and visible light, temperature, humidity, and oxidative degradation by atmospheric oxygen or metal ions [40]. In the gastrointestinal tract (GIT), enzymes degrade EOs, resulting in low concentrations reaching the gut, where pathogenic microbes are typically found [41]. These limitations reduce both the shelf life and antibacterial efficacy of EOs.

Encapsulation of EOs is a well-established strategy to mitigate intrinsic challenges such as volatility, chemical instability, and rapid degradation. Specifically, encapsulation techniques confer enhanced stability and prolonged shelf life by shielding EOs from environmental stressors, including light, oxygen, and heat, which otherwise promote oxidation and efficacy loss. Additionally, encapsulation diminishes the release of potentially harmful volatile compounds, thereby improving the safety profile of EOs [42].

Furthermore, encapsulation significantly enhances the bioavailability of EOs. The reduction in particle size and the application of protective coatings facilitate improved solubility and provide controlled release kinetics, preventing premature absorption and degradation within the gastrointestinal tract. This sustained release mechanism supports prolonged therapeutic or preservative activity while avoiding rapid fluctuations in concentration [43]. Encapsulation also enables targeted delivery and deeper tissue penetration, thereby augmenting the overall bioefficacy of EOs as natural preservatives or bioactive compounds [44].

In summary, encapsulation represents a strategic approach to enhance EOs stability, regulate release profiles, minimize toxicity, and improve bioavailability, effectively addressing numerous limitations associated with their direct application.

**Figure 3:**
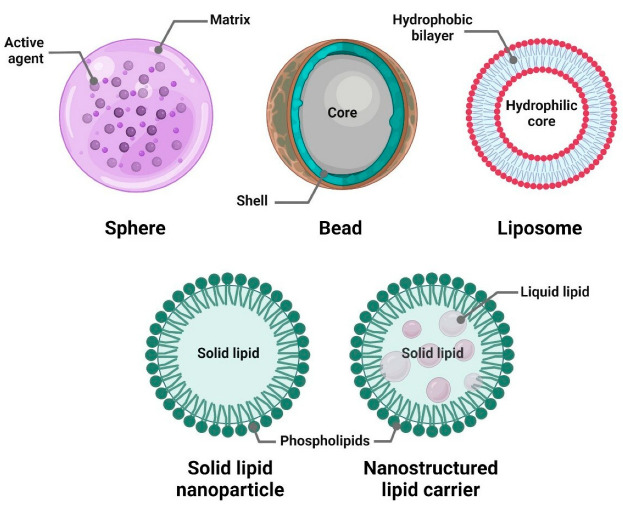
Structures of different types of particles based on natural matrices.

Encapsulation can formulate several types of particles (Figure 3) [45]. A uniform dispersion of an active compound within a continuous matrix forms a sphere [46]. A bead consists of a solid matrix in which the filler molecules are chemically bound to the matrix structure [47]. Liposomes are vesicles composed of one or more lipid bilayers encapsulating the active agent [48]. These delivery systems are widely applied in the pharmaceutical and nutraceutical industries [49,50,51,52].

In this review, we use the terms nano- and microparticles generically to refer to spherical particles with dimensions in the nanometer or micrometer range. The specific type of particle produced depends on the encapsulation method applied.

## 4. Methods of Encapsulation

**Figure 4:**
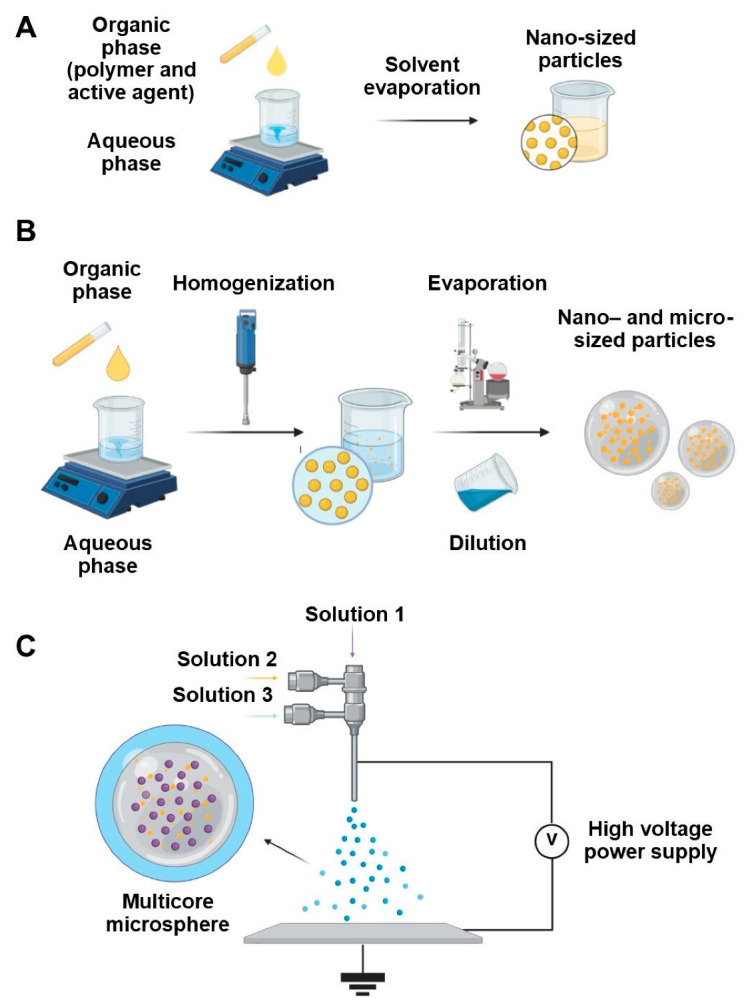
Methods of EOs encapsulation: (**A**) nanoprecipitation, (**B**) ESE and ESD techniques, (**C**) electrospray technique.

**Figure 5:**
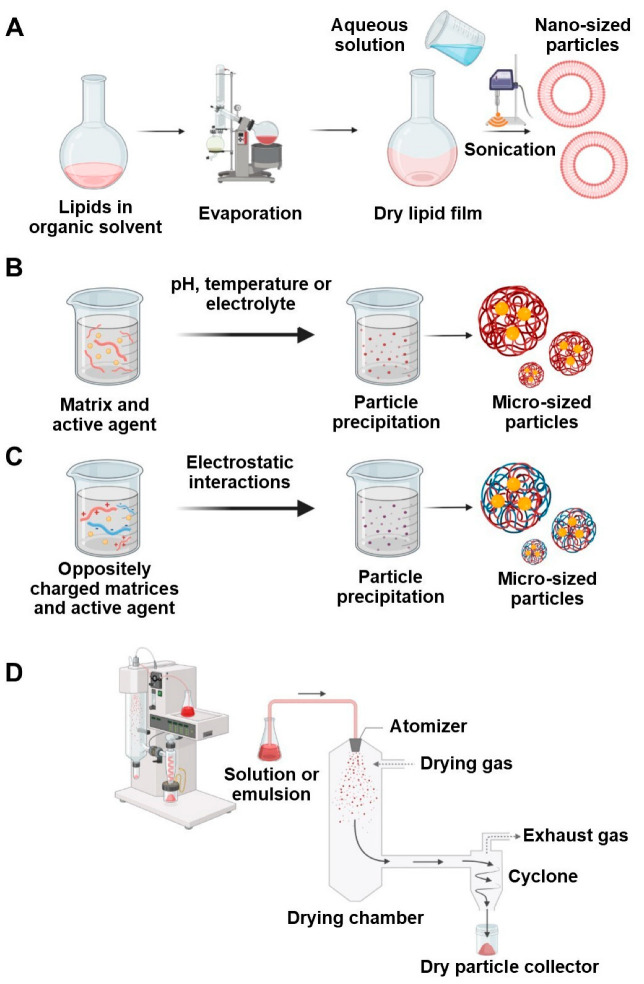
Methods of EOs encapsulation: (**A**) thin film hydration, (**B**) coacervation, (**C**) complex coacervation, (**D**) spray drying.

Currently, the literature provides plenty of methods for formulating particles. Below, we described the main methods used to obtain particles based on natural matrices loaded with EOs (Figure 4 and Figure 5).

### 4.1. Nanoprecipitation

Nanoprecipitation involves adding an immiscible organic phase, containing both the matrix material and the active ingredient, into an aqueous phase. Diffusion of the organic phase leads to nucleation and subsequent particle growth [53]. The method is reproducible and well scalable due to the use of specialized mixers [53]. The resulting particles typically range in size from 10 to 1000 nanometers.

### 4.2. Emulsification–Solvent Evaporation and Emulsion–Diffusion

Emulsification involves dispersing an organic solvent containing both the matrix material and the active ingredient in an immiscible liquid through electrostatic, hydrophobic, or hydrogen interactions between the matrix material and the active ingredient [54]. Shear stress, typically generated by an immersion homogenizer or ultrasound, breaks the matrix solution into microdroplets in the presence of a surfactant [55]. Two related methods differ based on how the organic solvent is removed: emulsion–solvent evaporation (ESE) and emulsion–diffusion (ESD). In the case of ESE, particles are formed due to the evaporation of organic solvent: as its concentration drops, polymer particles precipitate into the aqueous phase. In the case of ESD, particles are formed by dilution of the emulsion with water. The dilution leads to diffusion of organic solvent from the internal phase (globules) into the surrounding aqueous phase, leading to polymer aggregation and particle formation [56,57]. Both techniques are widely used for producing nano- and microspheres, particularly in laboratory settings. However, their scalability remains limited due to the energy-intensive emulsion homogenization [58,59,60,61,62].

### 4.3. Electrospray Techniques

Electrospray techniques, also known as electro-hydro-dynamic atomization, use an electric field to atomize a liquid into fine droplets, which then solidify into particles. The electric field force is generated between the needle and the platform where the particles are concentrated [63]. Depending on the type of needle and the number of possible fluid streams, electrospray systems can be classified as mono-coaxial (1 stream), coaxial (2 streams), and tri-axial (3 streams) electrosprays [64]. Electrospray techniques allow for the formulation of multilayered spheres with sizes ranging from 10 nanometers to 100 μm [63]. Although electrospray methods offer precise control over particle morphology and composition, they are difficult to scale up. The process relies on a delicate balance of forces, and small fluctuations in liquid flow, electric field strength, or emitter geometry can compromise droplet formation and stability. These challenges become more pronounced at an industrial scale [65]. Nevertheless, recent developments in specialized platforms and auxiliary equipment have improved scalability and process consistency [66].

### 4.4. Thin Film Hydration

Thin film hydration is the most common method for liposome preparation. Initial lipids are dissolved in an organic solvent and evaporated in a flask on a rotary evaporator. As a result, a thin lipid layer is formed on the walls of the flask. Next, the lipid layer is dispersed in an aqueous medium. Subsequent application of extrusion or homogenization (mechanical or ultrasonic) yields particles with sizes of 10–1000 nm [67]. Despite its simplicity, this method suffers from low throughput and low encapsulation efficiency, making it difficult to scale up [68].

### 4.5. Ionic Gelation

Ionic or ionotropic gelation relies on the ability of polyelectrolytes to covalently bind to each other in the presence of counterions to form hydrogel capsules. Ca^2+^ or triphosphate ions are commonly used due to their low cost and toxicity [69]. This method typically produces micro- and millimeter-sized particles. However, its scalability is limited by low throughput, as traditional nozzle systems produce capsules in a drop-by-drop manner. Using multiple nozzles can increase productivity [70].

### 4.6. Coacervation

Coacervation involves phase separation of the initial solution of matrix with the active ingredient to form two separate liquid phases—matrix-rich (coacervate) and matrix-depleted (equilibrium solution). Coacervate production occurs by particle precipitation due to reduced solubility of the polymer, often triggered by adding electrolytes, adjusting pH, or changing temperature [71].

In complex coacervation, two oppositely charged polymers interact electrostatically to form coacervate particles, which then precipitate similarly to simple coacervation [72].

To improve mechanical and thermal stability, the particles can be cross-linked using agents recognized as generally safe (GRAS), such as genipin [73], tannic acid or tannin [74], and transglutaminase [75].

Both types of coacervation typically produce micro-sized particles. While complex coacervation has gained attention for scale-up in the food industry [76,77,78], data on scalable simple coacervation methods remain scarce.

### 4.7. Spray Drying

Spray drying is based on moisture removal by rapid heating. The initial solution or emulsion is fed into the drying chamber using compressed gas in the form of small droplets. In the drying chamber, the hot gas is used to remove moisture at a temperature of 150–220 °C, forming dry particles that are collected in a tank [79]. The resulting powders may contain nano- (<1 µm), micro- (10–50 µm), or even larger particles (2–3 mm), depending on processing parameters [80]. Spray drying is highly reproducible and scalable with high yields [81]. However, the lack of a validated model for the mechanism of particle agglomeration during drying increases the time to select optimal conditions and may adversely affect the process efficiency [82].

The exploration of various encapsulation techniques for producing particles loaded with EOs reveals a diverse array of techniques, each with distinct advantages and limitations. Techniques such as nanoprecipitation, emulsification–solvent evaporation, electrospray, thin film hydration, ionic gelation, coacervation, and spray drying differ in terms of particle sizes, scalability potential, and operational complexities. The choice of encapsulation technique is critical and depends not only on the desired particle characteristics but also on the specific properties of the matrix material. Understanding these methods provides critical insights for researchers optimizing particle production for EOs encapsulation. In addition to technological aspects (scalability, auxiliary equipment, particle size), the choice of a particular method for encapsulation is determined by the properties of the particle matrix.

## 5. Wall Materials for EOs Encapsulation

Natural-origin matrices commonly used as wall materials for EOs encapsulation include polysaccharides (including gums), proteins, and lipids [11].

An ideal encapsulation matrix should protect the active compounds from degradation in both environmental and gastrointestinal conditions. It must be capable of efficient encapsulation, remain chemically inert to the active ingredients, and be odorless and tasteless. In addition, commercial availability and cost-effectiveness are essential considerations.

### 5.1. Polysaccharides

**Figure 6:**
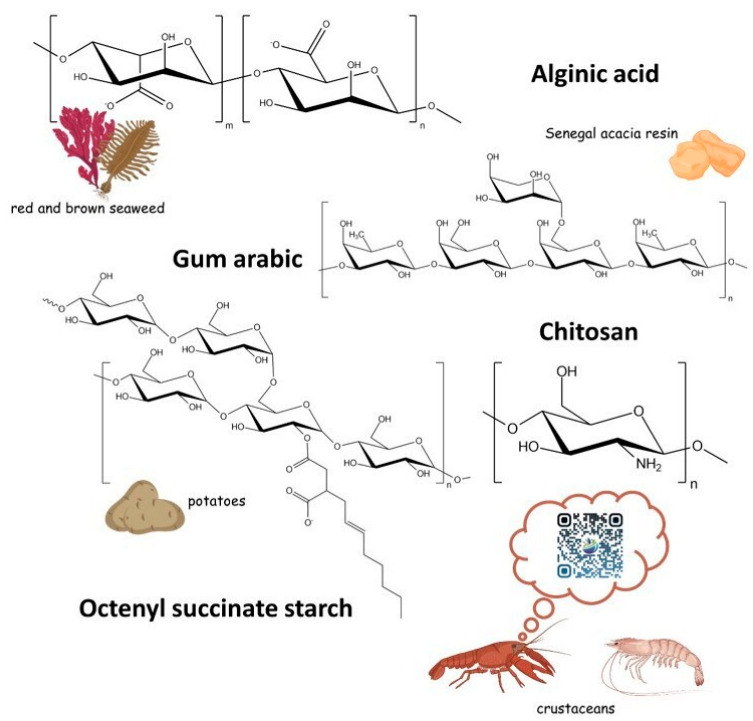
Polysaccharides as wall materials for EOs encapsulation.

Polysaccharides are a diverse group of naturally occurring high-molecular-weight carbohydrates. They consist of linear or branched polymer chains comprising monomers of sugar residues linked by glycosidic bonds [83]. Figure 6 shows the structures of the most widely used polysaccharides for Eos encapsulation: chitosan, alginate, starch, and gums [84,85].

The literature categorizes polysaccharides into starch and non-starch polysaccharides (NSPs), based on water solubility. Starch polysaccharides are typically water-insoluble, whereas NSPs form viscous aqueous solutions at room temperature [86]. In the food industry, NSPs are also referred to as industrial gums and include alginates, gum arabic, carrageenan, xanthan, etc. [87]. Although alginates are a subgroup of gums, they are discussed separately due to their unique encapsulation properties and particle synthesis methods.

#### 5.1.1. Chitosan

Chitosan, a linear polysaccharide derived from chitin via alkaline deacetylation, is approved by the U.S. Food and Drug Administration (FDA) for use as a feed additive in animal nutrition [88]. Toxicity studies indicate the high safety of chitosan. For instance, the oral LD50 for chitosan is 16 g/kg body weight in mice [89]. In another study [90], a single oral dose of chitosan oligomers up to 10 g/kg demonstrated a lack of clinical signs of toxicity in male and female Kunming mice.

Chitosan’s stability is pH-dependent, as it is prone to acid hydrolysis. After ingestion, partial enzymatic degradation begins in the oral cavity through lysozyme activity [91], and further metabolism occurs in the intestine. In the stomach’s acidic environment, the positively charged chitosan binds to negatively charged lipids such as cholesterol and phospholipids, forming complexes that are excreted in feces [92].

Chitosan hydrolysis products yield oligosaccharides that possess a number of beneficial properties, including anti-inflammatory, antibacterial, and improved growth performance [93].

The antibacterial activity of chitosan and its oligosaccharides results from multiple mechanisms targeting bacterial cell surfaces and intracellular components. In acidic conditions, protonation of chitosan’s amino groups facilitates electrostatic binding to negatively charged bacterial cell walls and membranes, disrupting membrane integrity and permeability. This leads to leakage of cellular contents and impaired nutrient transport, ultimately decreasing bacterial viability [94].

Additionally, chitosan forms a polymeric film on the bacterial surface, blocking nutrient uptake. Low-molecular-weight oligosaccharides penetrate bacterial membranes, interacting with intracellular targets such as DNA, thereby inhibiting transcription and protein synthesis. Interaction with metal ions on the bacterial surface further destabilizes the envelope, enhancing antibacterial effects. Synergistic action with metal nanoparticles like silver also potentiates membrane disruption [95].

The antimicrobial activity of chitosan is most pronounced in an acidic environment, where the polymer is soluble and exhibits a net positive charge.

Table 1 provides a summary of EO-loaded chitosan-based particles. Chitosan-based particles are primarily obtained through ionic gelation, with triphenyl phosphate (TPP) serving as the crosslinking agent. This technique yields chitosan particles with a diameter range of 40 to 1000 nanometers. The initial chitosan concentration significantly influences particle size. The degree of acetylation of chitosan represents the second significant factor influencing the characteristics of the resulting particles [96].

**Table 1:** Examples of EO-loaded chitosan particles.

Core Material	Wall Material	Method of Preparation	Properties of Particles	References
Cinnamon EO	Chitosan, β-cyclodextrin	Ionic gelation	Size 300–400 nm EE 40–60% Release 25%, 50% and 28% after 120 h (pH 7.0, 4.5, 12.0)	[97]
	Chitosan		Size 235.6 nm EE 40%	[98]
	Chitosan		Size 0.1–1 µm EE 56–70% Release 25–40% after 400 min	[99]
Bitter orange oil	Chitosan	Ionic gelation	Size 40–60 nm EE 5–15% Release 20% after 20 days (pH 7.0)	[100]
Cumin seed oil	Chitosan	Ionic gelation	Size 150–250 nm EE 27% Release 69%, 61%, 30% and 48% after 5 h (pH 3, 5, 7, and 11)	[101]
Jasmine EO	Chitosan, pectin	Ionic gelation	Size 500–700 nm EE 8–30% Release 50% after 48 h (pH 7.4)	[102]
Achillea millefolium EO	Chitosan	Ionic gelation	Size 85–145 nm EE 85–90%	[103]
Cardamom EO	Chitosan	Ionic gelation	Size 50–100 nm EE 90%	[104]
Clove EO	Chitosan	Ionic gelation	Size 223–444 nm EE 55–70% DSC decomposition of free EO at 124 °C, decomposition of encapsulated EO at 320 °C	[105]
			Size 100 nm EE 30–45% Release 30% after 60 days (pH 3 and 5)	[106]
Coriander EO	Chitosan	Ionic gelation	Size 57–80 nm EE 27–78% Release 90% after 175 h in PBS media	[107]
		Spray drying	Size 400 nm–7 µm EE 5–25% Release 60% after 336 h in PBS media TGA EO decomposition at 100–200 °C, chitosan decomposition at 200–350 °C	[108]
Thyme EO	Chitosan	Nanoprecipitation	Size 10 nm EE 70% Release 100% after 360 min in water	[109]
Oregano (*Origanum vulgare)* EO	Chitosan	Ionic gelation	Size 407 nm EE 83%	[110]
Nettle (*Urtica dioica* L.) EO	Chitosan	Ionic gelation	Size 208–369 nm EE 59–68%	[111]
Clove (*Eugenia caryophyllata*) EO	Chitosan	Ionic gelation	Size 148–1287 nm EE 31–45% Release 30% after 56 days (pH 3)	[106]

A literature review revealed no consistent correlation between particle size and encapsulation efficiency. In general, encapsulation efficiency falls between 30% and 70%, relating also to the physicochemical properties of the encapsulated EOs.

Chitosan matrices can enhance the thermal stability of EOs. Some studies showed that encapsulated EOs remain stable at temperatures 25–50% higher than those of their free derivatives. Thermogravimetric analysis (TGA) indicates that chitosan itself degrades between 200 and 350 °C [108].

Assessing the influence of particle parameters on the release rate of encapsulated EOs is challenging due to the lack of standardized experimental protocols. However, among studies conducted under comparable conditions, a clear trend emerges: in acidic environments, release rates are approximately twice as fast as in neutral media [97,101].

#### 5.1.2. Gums

Gums are complex polysaccharides or their derivatives that form gels, highly viscous mixtures, or solutions when dispersed in cold or hot water. Gums can be classified into five categories: seed gum (guar gum, Balangu seed gum), plant exudates (gum arabic), microbial exudates (xanthan, dextran), seaweed extracts (alginates, carrageenan), and synthetic gum derived from cellulose (carboxymethyl cellulose, methyl cellulose) [112].

Gum arabic is one of the most commonly used matrices for EOs encapsulation. Although indigestible in both humans and animals, it has been recognized as a safe dietary fiber by the FDA since the 1970s [113]. In animal nutrition, gum arabic also functions as a natural prebiotic, contributing to improved intestinal immunity and liver metabolism. The oral administration of gum arabic at a dose of 0.1 g/kg body weight enhances renal function in rats with chronic kidney disease [114].

EOs are typically encapsulated in gums using ultrasound-assisted emulsification, followed by spray or freeze-drying (Table 2). The resulting particles range from nano- to microscale. The encapsulation efficiency varies widely (16–89%) and does not show a direct correlation with particle size.

**Table 2:** Examples of EO-loaded gum particles.

Core Material	Wall Material	Method of Preparation	Properties of Particles	References
Ginger EO	Cashew gum	Spray drying	Size 5–30 μm EE 28% TG matrix decomposition at 250 °C; free EO evaporation below 180 °C	[115]
	Cashew gum–Inulin		Size 4–33 μm EE 16–31% TG matrix decomposition at 250 °C; free EO evaporation below 180 °C	
Lemongrass EO	Gum Arabic– Maltodextrin– OSA-starch	Spray drying	Size 5–13 μm EE 55–81% TGA free EO evaporation at 40–150 °C; matrix decomposition at 200–350 °C	[116]
Peppermint flavor	Gum Arabic	Spray drying	Size 45–256 nm EE 46–88%	[117]
*Mentha longifolia* L. EO	Balangu seed gum	Electrospraying	Size 96 nm EE 82–88% Release approx. 100% after 180 min in aqua media DSC complete EO decomposition at 169 °C; matrix decomposition at 291–345 °C; capsule decomposition at 223 °C	[118]
Fish oil Garlic EO	Persian gum–Chitosan	Electrostatic layer-by-layer deposition	Size 23–152 nm EE 63–86% DSC decomposition of matrix and capsule at 250–270 °C	[119]
Rosemary EO	Cashew gum galactomannan	Spray drying	EE 74–87%	[120]
Sweet basil EO	*Lepidium sativum* and *Lepidium perfoliatum* seed gums	Emulsification	Size 331–592 nm EE 77–89%	[121]
D-limonene	Alyssum homolocarpum seed gum	Electrospraying	EE 78–81% TGA free d-limonene decomposition at 150 °C; particles’ decomposition at 230 °C	[122]

Studies evaluating EO release from gum-based matrices in simulated gastric (SGF) and intestinal fluids (SIF) remain limited. In one study, authors showed that a single gum matrix can achieve rapid release with approximately 90% of the EOs released within 10 min in an aqueous medium [118].

The thermal analysis results indicate an improvement in the stability of encapsulated EOs within gum matrices. In some cases, combining gums with other polysaccharides, such as chitosan, significantly enhances the thermal stability of the particles [119]. For example, de Barros Fernandes and coauthors showed the importance of selecting the appropriate polysaccharide. They found that the inulin–cashew gum co-formulated matrix lacked an increase in EOs thermal stability. When heated to 250 °C, the degradation rate of cashew gum-based particles was found to be under 20%, while for cashew gum–inulin particles, the degradation rate ranged from 23% to 29% [115].

#### 5.1.3. Alginate

Alginates are a natural anionic polymer commonly derived from the brown algae *Phaeophyceae*. They are salts of alginic acid. The structure of alginates is linear and is represented by D-mannuronate (M) and L-guluronate (G) residues connected by 1,4-glycosidic bonds (Figure 6). The ratio of M and G blocks varies in alginates extracted from different sources. The mechanical strength, porosity, and resistance to salts and chelating agents of alginate-derived beads are directly proportional to the proportion of G blocks present [123].

In the food industry, alginates are classified as GRAS 21 CFR 295 Regulations 184.1133, 184.1610, 184.1724, 184.1187. Within the human body, these polymers stay intact and are excreted through the intestinal tract. Although stable under neutral conditions, alginates are sensitive to pH shifts and may degrade through alginate lyases secreted by certain gut microbiota [124]. Subchronic toxicity studies of sodium alginate in rodents showed a lack of adverse effects at a dose of 13.5 mg/kg/d in a 90-day study [125].

The main method for producing alginate beads is ionic gelation (Table 3). In practice, the size of particles with different EOs ranges from a few microns to several millimeters. The method generally yields high encapsulation efficiency (>70–80%).

**Table 3:** Examples of EO-loaded alginate beads.

EOs	Wall Material	Method of Preparation	Properties of Particles	References
*Perilla frutescens* (L.) *Britt.* EO	Alginate	Ionic gelation	EE 57% Release 80% and 30% after 24 h (25 °C and 4 °C) DTG alginate matrix decomposition at 210–320 °C TG-free EO evaporation at 50–200 °C	[126]
Cinnamon EO	Alginate	Ionic gelation	Size 2.44 mm EE 85%	[127]
Thyme EO	Alginate	Ionic gelation	Size 890 µm EE 85%	[128]
Cumin EO	Alginate	Ionic gelation	Size 2.1 mm LC 0.22% Release 96% after 180 min in SGF and 10% after 180 min in SIF	[129]
Clove EO	Alginate	Emulsification	Size 1.5–3.0 mm EE 24% Release 50% after 240 min (pH 7)	[130]
Lavender (*Lavandula angustifolia*), tea tree (*Melaleuca alternifolia*), bergamot (*Citrus bergamia*), and peppermint (*Mentha piperita*) EOs	Alginate	Electrostatic extrusion	Size 0.9–1.2 mm	[131]
Tea tree EO	Chitosan and Alginate as external wall Methyl cellulose as internal wall	Spray drying	Size 7–11 µm EE 90% Release 60–90% after 72 h in PBS media	[132]
Carvacrol	Pectin–Alginate	Spray drying	Size 1.96 µm EE 77% Release 60% after 3 h in PBS media	[133]
Cinnamon EO	Alginate	Spray drying	Size 2 µm EE 88.1% Release 21% after 360 min in PBS media	[134]

Azad and coauthors demonstrated the distribution pattern of alginate beads in the GI tract of rats [135]. One hour after administration, the majority of the beads were located in the stomach. After three hours, the beads were observed in the lower small intestine, where the release of encapsulated black seed oil occurred (complete erosion was detected 6 h after administration) [135]. These findings highlight alginate’s potential to target EOs delivery to absorption-relevant regions of the GI tract.

Li and colleagues revealed that the temperature at which approximately 50% of the alginate matrix undergoes degradation was 225 °C [126], and the TGA profile of the encapsulated EO exhibited a slower weight loss, reaching only 19% compared to the free EO. These findings highlight the effectiveness of using alginate beads to reduce the volatility of EOs.

In another work, alginate beads significantly improved EOs’ stability during storage: the concentration of tea tree EO within the beads showed only a 0.1% decrease after one month of storage, while the structure of the beads remained unchanged for two weeks [132].

Researchers use alginates both as a single matrix material and in combination with other biopolymers. Blended matrices can improve alginate stability and increase EOs encapsulation efficiency [136]. Currently, alginate–protein (see Section 5.2) and alginate–polysaccharide composites are preferred for oral delivery applications [133].

#### 5.1.4. Starch

Starch’s hydrophobic nature makes EO encapsulation challenging without toxic solvents. The chemical, enzymatic, or physical modification of the starch molecule yields derivatives with improved water solubility. For instance, β-cyclodextrin is a product of the enzymatic hydrolysis of starch, while maltodextrin and octenylsuccinate (OSA) starch can be obtained by enzymatic or acid hydrolysis [137].

In monogastric animals, starch is digested by salivary and pancreatic α-amylases in the oral cavity (pH 6.7–7) and small intestine (pH 6–7), yielding maltose, maltotriose, and α-predominant dextrins, respectively. These products then undergo hydrolysis, leading to glucose formation. In ruminants, microbial enzymes first degrade starch in the rumen, with residual fractions digested in the small intestine [138]. Modified starch (e.g., OSA) shows slightly reduced digestibility compared to native starch [139].

The European Food Safety Authority approved the application of starch sodium octenyl succinate (E 1450) as a food additive for infants up to 16 weeks of age and for all population groups [140]. Toxicological studies report no adverse effects on systemic or reproductive health of aluminum starch octenylsuccinate or its sodium salt [141].

Ultrasound-assisted emulsification, nanoprecipitation, and spray drying are commonly used for encapsulating EOs into starch-based particles (Table 4). Particle sizes range from 20 to 4000 nm, with encapsulation efficiencies between 45% and 95%.

**Table 4:** Examples of EO-loaded starch particles.

EOs	Wall Material	Method	Particle’s Properties	References
Peppermint EO	Short linear glucan debranched from waxy maize starch	Ultrasound-assisted emulsification	Size 20 nm EE 75–88% Release 27–33% after 150 min in aqua media (80 °C) DSC melting of the particles at 84–110 °C	[142]
Menthone, oregano, cinnamon, lavender, and citral EO	Short linear glucans debranched from waxy maize starch	Nanoprecipitation	Size 93–113 nm EE 87% Release 85% after 48 h in PBS media	[143]
Lemongrass EO	OSA-starch	Spray drying	Size 13 µm TGA oil evaporation at 50–150 °C, particle decomposition at 200 °C	[116]
Orange EO	Short linear glucans debranched from rice starch	Spray drying	Size 30–40 µm EE 57–99%	[144]
Rosemary EO	OSA-starch	Electrospraying	EE 82–98%	[145]
*Rosmarinus officinalis* and *Zataria multiflora* EOs	OSA-starch	Spray drying	Size 8–11 µm EE 5–52% Release 90% after 30 d in the atmosphere (27 ± 3 °C and 70–75% relative humidity)	[146]
Vanilla EO	Jackfruit seed starch	Ultrasound-assisted emulsification	EE 79%	[147]
Rose EO	(OSA)-modified starch and maltodextrins (MDs)	Homogenizer-assisted emulsification	Size 2 µm EE 45% TGA decomposition of the particles at 235–309 °C, oil evaporation at 275 °C	[148]
Lemon EO	Chitosan and modified starch (Hicap 100)	Ultrasound-assisted emulsification	Size 339–553 nm EE 85% DSC free oil evaporation at 74–124 °C, decomposition of the particles at >200 °C	[149]

Starch-based particles enhance the thermal stability of EOs when the evaporation/degradation temperature is below 200 °C. The results of TGA studies showed that the onset temperature of degradation of porous starch is approximately 200 °C [150]. In the case of OSA-starch, the degradation onset temperature was at 150–200 °C [116,148].

These matrices also protect encapsulated EOs from oxidation, decreasing peroxide formation and increasing oxidative stability [147,151]. For example, orange EO encapsulated in starch showed no oxidative change over 28 days [144], and vanilla EO encapsulated in jackfruit starch retained stability for 160 days at 20 °C [147].

In vitro release studies mimicking the human body remain scarce. Qiu and colleagues showed that menthone EO released from short glucan chain nanoparticles in PBS (pH 7.4) reached 50% release in 10 h, followed by a plateau phase and 85% in 48 h [143]. Additionally, heating the aqueous release medium to 80 °C led to a partial release of peppermint EO from the short glucan chain matrix, ranging from 27% to 33% over 150 min [142].

### 5.2. Proteins

EOs have been successfully encapsulated in particles made from both animal-based proteins (e.g., whey, gelatin) and plant-based proteins (e.g., soy, zein) [152,153].

In monogastric animals, the digestion of proteins occurs in the stomach by pepsin and hydrochloric acid. The resulting amino acids are then absorbed in the small intestine. In ruminants, some proteins are metabolized in the rumen by microbiota, while others are digested and absorbed in the small intestine [154].

Common techniques for the synthesis of EO-loaded particles based on protein are complex coacervation and spray drying (Table 5). Spray drying yields particles within the nano- and micro-size ranges, achieving an encapsulation efficiency of 65% to 95%. For complex coacervation, particle sizes generally fall within the tens to hundreds of microns, and EOs encapsulation efficiency frequently surpasses 75%.

**Table 5:** Examples of EO-loaded protein particles.

EOs	Wall Material	Method	Particle’s Properties	References
Tuna oil and Mint (*Mentha piperita*) EO	Whey protein isolate-inulin	Spray drying	Size 190–280 nm EE 94%	[155]
Lime EO	Whey protein concentrate-maltodextrin	Spray drying	Size 3–4 µm EE 67–83% Release 60% after 150 min in mineral oil media TGA decomposition of the particles at 250 °C	[156]
Eugenol	Whey protein isolate- Maltodextrin	Spray drying	Size 0.1–10 µm EE 94–99% TGA free oil evaporation at 200–250 °C, decomposition of the particles at 283 °C	[157]
Chia EO	Whey protein concentrate–Mesquite gum or gum Arabic	Spray drying	Size 13–28 µm EE 70–81%	[158]
Black pepper (*Piper nigrum* L.) EO	Gelatin–Sodium alginate	Complex coacervation	EE 49–82%	[159]
Black pepper (*Piper nigrum* L.) EO	Lactoferrin–Sodium alginate	Complex coacervation	EE 32–85% Release 24% for 2 h in SGF media after following 85% for 2 h in SIF media	[160]
Shiitake (*Lentinula edodes*) EO	Gelatin–Carboxymethylcellulose	Complex coacervation	EE 86%	[161]
Citronella EO	Gelatin–Sodium alginate	Complex coacervation	Size 434 µm EE 74% TGA core material evaporation at 230–270 °C	[162]
Thyme EO	Soy protein–Alginate	Atomization via electrostatic extrusion	Size 0.6–1.4 mm EE 72–80% Release 42–55% after 60 min in SGF media (37 °C), 90–100% after following 60 min in SIF media	[136]
Carvacrol	Whey protein–Alginate	Extrusion	Size 250 and 800 µm Release 4.5% (for 250 µm) or 1.3% (for 800 µm) after 1 h in SGF media, 100% after following 4 h (250 µm) and 5 h (800 µm) in SIF media	[163]
Cinnamaldehyde	Gelatin–Pectin	Complex coacervation	Size 80–98 μm EE 85–89% Release 22% after 20 min in aqua media (80 °C) TGA core material evaporation at 125 °C and degradation at 225 °C; capsule decomposition at 250–400 °C	[164]
Rose EO	Mung bean protein Isolate–Pectin	Complex coacervation	Size 15 μm EE 90% Release 35% after 2 h in SGF media and 80% after 2 h in SIF media TGA: core material evaporation below 100 °C, capsule decomposition at 200–500 °C	[165]
Cardamom EO	Whey protein–Alginate	Internal gelation	Size 100 µm EE 84% Release 100% after 70 min in artificial saliva media	[166]

Besides the encapsulation technique, the EOs type influences the size of protein-based particles. Da Silva and Pinto noted that EOs properties influence the viscosity and surface tension of the formulation, which, in turn, alters particle size [167].

Due to their pH sensitivity and low solubility below pH 7, proteins are rarely used alone. They are often combined with polysaccharides to form more stable, multicomponent matrices [168,169]. Multicomponent matrices enhance protein matrix stability within the physiological environment, thereby ensuring the sustained release of encapsulated EOs.

In vitro studies demonstrated the potential for the development of a system with slow release in SGF medium (up to 25% in 2 h) and subsequent almost complete digestion in SIF medium (Table 5). This profile results in EOs accumulation in the lower intestinal tract, which is the location of pathogenic bacteria, and ensures prolonged antibacterial activity.

EOs encapsulation in protein–polysaccharide matrices enhances the thermostability of EOs. TGA studies revealed the enhanced stability of citronella EO in gelatin–alginate particles. The evaporation temperature of citronella EO in particles was 230 °C, while unprotected citronella EO decomposed at 201–207 °C [162]. Talón et al. showed enhanced thermostability of eugenol-loaded whey protein–maltodextrin–chitosan particles: the evaporation temperature of encapsulated eugenol was 200–250 °C compared to 175 °C for the free compound [157]. Campelo and colleagues also confirmed the stability of the whey protein–maltodextrin system up to 200 °C [156]. These results indicate that encapsulating EOs could be an effective approach to prolonging their shelf life.

### 5.3. Lipid-Based Systems

Lipid-based encapsulation systems include traditional liposomes consisting of an amphiphilic lipid bilayer and a hydrophilic core, relatively new solid lipid nanoparticles (SLN), and nanostructured lipid carriers (NLC) [170]. SLN capsules or spheres are composed of solid lipids at room temperature. In contrast to SLN, NLC is a shapeless matrix in which the lipid phase contains both solid (fat) and liquid (oil) lipids at ambient temperature (Figure 3). Classical liquid liposome membrane components (phospholipids and sterols), along with solid (fatty acids, steroids, waxes, glycerides) lipids of SLN and NLC constituents, are mainly metabolized by pancreatic lipases in the small intestine [171].

Phospholipids such as soy lecithin, commonly used for liposome production, demonstrated minimal toxicity in acute oral studies, short-term oral studies, and subchronic dermal studies in animals. Additionally, lecithin lacked reproductive toxicity and mutagenic properties [172].

The primary techniques for formulating liposomes, SLN, or NLC are thin film dispersion and high-pressure homogenization, respectively (Table 6). The particles obtained by these methods typically range in size from 10 to 1000 nm. But, the encapsulation efficiency is generally moderate and often falls between 30% and 70%.

**Table 6:** Examples of EO-loaded lipid particles.

Core Material	Wall Material	Method of Preparation	Properties of Particles	References
Chrysanthemum EO	Single-layer liposomes–Soy lecithin and cholesterol	Thin film hydration method	Size 97–232 nm EE 17–50% Release 37%, 48%, 71%, 88% after 30 min in the air media (at 4 °C, 12 °C, 25 °C, 37 °C, respectively)	
	Double-layer liposomes–Soy lecithin and cholesterol + chitosan	Layer-by-layer electrostatic deposition method	Size 530–793 nm EE 43% Release 25%, 33%, 48%, 60% after 30 min in the air media (at 4 °C, 12 °C, 25 °C, 37 °C, respectively)	[173]
	Triple-layer liposomes–Soy lecithin, cholesterol, and chitosan + pectin	c	Size 642–3236 nm EE 43% Release: 12%, 17%, 22%, 25% after 30 days in the air media (at 4 °C, 12 °C, 25 °C, 37 °C, respectively)	
Thyme EO	Soy lecithin and cholesterol	Thin film dispersion method and	Size 182 nm EE 35% Release 26% after 15 days in the air media	[174]
*Eucalyptus citriodora* EO	Soy lecithin and cholesterol	Thin film dispersion method	Size 150–295 nm EE 22% Release 39% and 68% after 28 days in the air media (at 4 °C and 25 °C, respectively)	[175]
*Oliveria decumbens* EO	Phosphatidylcholine	Thin film dispersion method	Size: 168 nm Release 100% after 4 days in aqua media	[176]
*Zataria multiflora* EO	Glyceryl mono stearate and Precirol^®^ ATO5	High-shear homogenization and ultrasound methods	Size: 255.5 nm PDI 0.369 EE 84%	[177]
*Zataria multiflora* EO	Stearic acid	High-pressure homogenizer method	Size: 134 nm PDI 0.24 EE 64.6%	[178]
*Eugenia caryophyllata* EO	Glyceryl mono stearate	High-shear homogenization and ultrasound method	Size 1231 nm PDI 0.384 EE 69.17%	[179]
Bergamot EO	Precirol^®^ ATO5	High shear homogenization	Size 194.70–437.50 nm PDI 0.17–0.70 Release 57–100% after 24 h in PBS pH 7.4	[180]
		Solvent diffusion	Size 133.90–365.10 nm PDI 0.18–0.39	
*Cuminum* *cyminum* L. EO	Cocoa Butter and Cacao Butter Substitute	High shear homogenization method and ultrasonic application	Size 86.11–129.53 nm PDI 0.1–0.25 EE 80.12–92.15%	[181]
Clove EO	Carnauba wax and beeswax	Low-energy nanoemulsification method coupled with high shear homogenization and sonication	Size 121–1489 nm PDI 0.08–0.31 EE 58–66%	[182]
*Ziziphora clinopodioides Lam.* EO	Precirol^®^ ATO5 and Compritol^®^ 888 ATO	High-shear homogenization and ultrasound	Size 241.1 nm PDI 0.312 EE 93%	[183]
Yuxingcao EO	Compritol^®^ 888 ATO	High-shear homogenization	Size 171.2–811.9 nm PDI 0.260–0.287 EE 76.61–90.20%	[184]
Frankincense and myrrh EO	Compritol^®^ 888 ATO	High-pressure homogenization	Size 113.3 nm EE 80.60%	[185]
*Rosmarinus officinalis* EO	Glyceryl tristearate	Ultrasonication	Size 103 nm EE 51.2%	[186]
Citral EO	Imwitor^®^ 900 K	High-pressure homogenization	Size 97.7 nm PDI 0.249	[187]
*Nigella sativa* L. EO	Softisan^®^154 and *N. sativa*	Hot homogenization	Size 66.27–142.70 nm PDI 0.18–0.27	[188]
Cinnamon EO	Cocoa butter	High-shear homogenization	Size 100–120 nm EE 82.1%	[189]

Liposomes exhibit relatively slow in vitro digestion. Lin et al. found that the digestibility level of liposomes loaded with thymol was 20–30% in SGF medium after 1 h and 30–40% in SIF medium during the following hour [174].

Long-term stability studies show that EO-loaded liposomes remain structurally intact for up to 28 days with Eucalyptus citriodora EO [175] and up to 60 days with thyme EO when stored at 4 °C [174]. Regarding the stability of phospholipid-derived particles, it is essential to consider another factor: the oxidation of phospholipids. This oxidation process is driven by reactive oxygen species, which modify the phospholipids [190]. The oxidation of phospholipids within liposomes leads to physical changes in the liposomal membrane, increasing the risk of oxidation of encapsulated components [191].

The assessment of lipid peroxidation typically involves the measurement of secondary oxidation products, such as malondialdehyde (MDA). Lin et al. synthesized triple-layer liposomes in which the concentration of MDA remained low (4–10 ng/mL) for 15 days during 4 °C and 12 °C storage, while MDA content for single and double-layer liposomes ranged from 10 to 40 ng/mL under the same conditions [173]. Temperature increase led to an increase in the rate of phospholipid oxidation: MDA concentration reached 14 ng/mL at 25 °C and 18 ng/mL at 37 °C for triple-layer liposomes, 45 and 35 ng/mL at 25 °C, and 55 and 45 ng/mL at 37 °C for single and double-layer liposomes, respectively. Other studies [174,175] demonstrated that β-cyclodextrin, as a cryoprotectant at high concentrations, effectively protected the phospholipid membrane from oxidative damage.

In conclusion, the encapsulation of EOs using various natural matrices such as polysaccharides, proteins, and lipids presents a promising approach to enhance the stability, bioavailability, and controlled release of these volatile compounds. In this part, we emphasized the significant role of polysaccharide matrices like chitosan, alginate, and starch, which demonstrate varying degrees of encapsulation efficiency and thermal stability. Additionally, the utilization of proteins and lipid-based systems further diversifies the strategies for EOs encapsulation, offering tailored properties for specific applications in food, pharmaceuticals, and nutraceuticals. The findings emphasize the importance of selecting appropriate encapsulating materials and methods, as well as the need for standardized protocols to evaluate the release dynamics and stability of encapsulated EOs under physiological conditions.

EE exhibits considerable variability due to the interactions between EOs and the matrix, as well as the influence of diverse processing parameters [192]. Also, EOs’ volatility directly contributes to losses of EE as constituents with higher volatility exhibit increased susceptibility to evaporation or chemical degradation during processing, particularly under conditions of elevated temperature or extended processing duration [193]. Careful selection of the matrix, assessment of compatibility, optimization of process design, and stringent control of environmental conditions are critical for improving EE in practical applications.

Future research directions could focus on optimizing encapsulation techniques, exploring the synergistic effects of combined matrices, and conducting in vivo studies to better understand the behavior of encapsulated EOs in biological systems.

## 6. Considerations for Wall Material and Encapsulation Technique Selection

**Figure 7:**
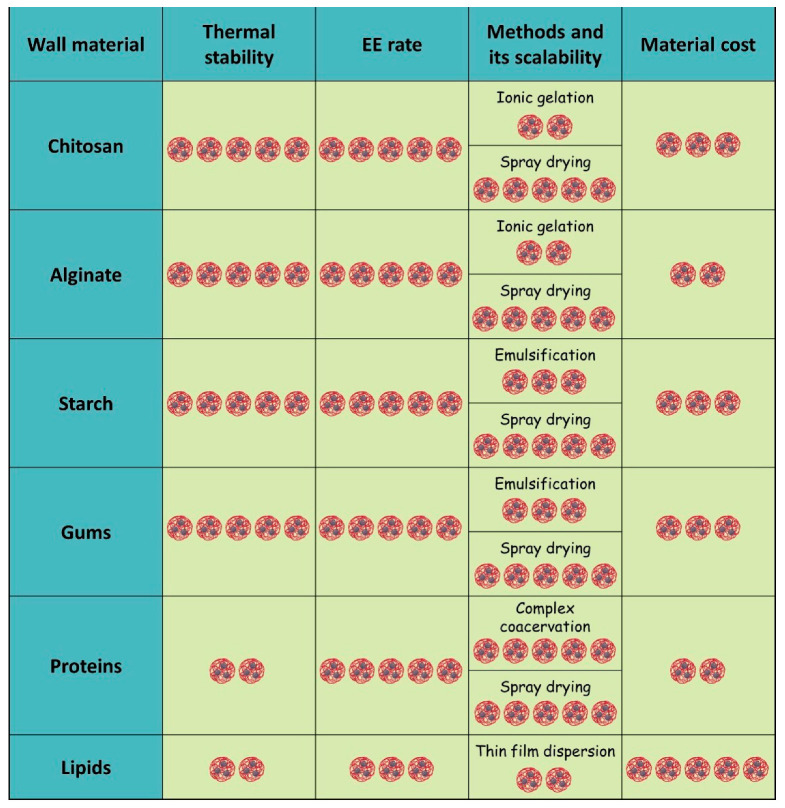
Comparison of parameters for matrices derived from natural sources. The pricing data is based on analyses of costs calculated per 100 g of product, as detailed on the Sigma-Aldrich website [197]. One represents the lowest level of the trait. Five represents the highest level.

This section summarizes key findings from Section 3 and Section 4, highlighting critical factors for selecting suitable matrix formulations and encapsulation techniques for EOs. We evaluated thermal stability, encapsulation efficiency, matrix cost, and method scalability (Figure 7) to provide a structured comparison of the advantages and limitations associated with each matrix type and technique.

Thermal stability is a crucial characteristic of particles, influencing shelf life and determining whether particles can be incorporated into feed pellets. Pelletization plays a crucial role in animal nutrition, as it improves feed density, enhances nutritional uniformity, and reduces ingredient segregation during transport and consumption [194]. Moreover, pelletizing minimizes selective feeding behavior in animals, ensuring more consistent nutrient intake [195]. Among the various matrices considered, lipid and protein matrices exhibit the lowest thermal stability. In contrast, polysaccharide-based particles, made from chitosan, gums (including alginates), and starch, demonstrate high thermal stability levels, with degradation onset temperatures around 200 °C. Utilizing combined matrices like chitosan–gum, starch–protein, or starch–maltodextrin can significantly enhance the thermal stability of these particles [119,148,196].

Encapsulation efficiency (EE) is another critical parameter, reflecting both technological performance and economic feasibility. A higher percentage of encapsulation efficiency indicates a more advantageous production and application of the particles. Most natural matrices discussed in this review demonstrate high and comparable EE values, with the notable exception of lipid-based systems, which typically show lower encapsulation efficiencies.

Regarding encapsulation techniques and scalability, spray drying emerges as the most convenient, highly scalable, and cost-effective technique for obtaining particles based on natural matrices, including combined matrices. Additionally, for combined matrices that include proteins, complex coacervation is also commonly used.

Cost is a decisive factor in selecting a matrix for commercial applications. Proteins and alginates stand out as the most economical options among the matrices discussed. The price of proteins fluctuates significantly based on their source and extraction method. Our analysis focuses on the most commonly used proteins, such as gelatin and soy protein. Chitosan, starch, and gum arabic, all widely used in EOs encapsulation, are moderately priced, typically higher than alginates and proteins, though still within an acceptable range for industrial use. The costs of chitosan and starch can vary considerably depending on their sources and any modifications. Lipid-based components, particularly those used in liposome production (e.g., cholesterol, lecithin), tend to be significantly more expensive. Cholesterol, for example, can cost an order of magnitude more than lecithin, and both are required in liposomal formulations.

The encapsulated systems demonstrate strong sustainability profiles, combining effective biodegradability with environmental safety to support sustainable pest and disease management in agriculture.

Chitosan and alginate are naturally derived polysaccharides known for their high biodegradability and biocompatibility. Alginate forms various biodegradable structures such as hydrogels and microcapsules, degrading efficiently in biological environments without harmful residues [198]. Chitosan undergoes enzymatic degradation facilitated by its amine and hydroxyl groups, promoting environmental breakdown and reducing persistence in soils [199]. These polymers yield non-toxic degradation products assimilated into natural biogeochemical cycles, minimizing ecological accumulation and environmental risks associated with synthetic materials.

Sustainability is further supported by the renewable biomass origins of these polymers—chitosan from crustacean shells and alginate from algae—ensuring low environmental impact during production. Similarly, gums, starch, proteins, and lipid-based nanoparticles share natural sources and biodegradability, aligning with eco-friendly agricultural practices. Life cycle assessments indicate that these delivery systems enable controlled release of essential oils, reducing required doses and off-target contamination, while degrading into harmless metabolites that prevent toxic waste buildup [200].

In summary, composite (mixed) matrices represent a promising trend in EOs encapsulation. These multicomponent systems combine the benefits of individual matrix types, offering enhanced thermal and oxidative stability, improved release profiles, and often, lower production costs.

## 7. Application of Encapsulated EOs in Livestock Animals

Numerous in vivo studies have demonstrated the efficacy of encapsulated EOs, focusing primarily on their antibacterial activity, influence on growth performance, and biochemical blood parameters in livestock animals.

The improvement in growth performance is closely linked to enhanced feed digestibility. EOs stimulate gastrointestinal function by increasing the activity of key digestive enzymes—trypsin, lipase, and amylase—as well as promoting gastric secretion. Moreover, EOs enhance the volume of gastric secretion. Collectively, these effects increase the absorption of nutrients, thereby enhancing animal performance [201,202].

### 7.1. Antibacterial Effect of Encapsulated EOs in Livestock Animals

Table 7 summarizes the available data on the impact of encapsulated EO components on gut microbiota in the most farmed monogastric animals—poultry and pigs. One study reported that a blend of mint, thyme, and cinnamon EOs, along with thymol encapsulated in a chitosan matrix, has been observed to exert notable antibacterial effects against *E. coli* (Table 7). *E. coli* are opportunistic bacteria that are typically present in the intestines of poultry and a majority of other animals. The proliferation of these bacteria is typically regulated by other bacterial species. However, the formation of large colonies can result in significant adverse effects, including disease and even mortality [203].

In the case of *C. perfringens*, a major cause of necrotic enteritis in poultry, the use of a carvacrol monocomponent in a chitosan matrix resulted in an insignificant decrease in bacterial growth. The utilization of a lipid matrix with an encapsulated mixture lacked any effect on bacterial growth. The use of polysaccharide matrices encapsulating both the mixture and individual EOs (citral EO) demonstrated a notable reduction in the growth of *C. perfringens* [204]. Necrotizing enteritis represents the most prevalent disease affecting poultry, with an estimated economic impact of USD 6 billion per year on the global poultry industry [205].

Interestingly, the bactericidal effect of EOs against probiotic bacteria (*Lactobacillus* spp. and *Bifidobacterium* spp.) demonstrates selectivity of action in some cases. The use of polysaccharide-based particles loaded with a blend of EOs demonstrated a notable positive impact on *Lactobacillus* spp. while concurrently inhibiting the growth of *E. coli* and *C. perfringens* in broilers [206,207,208,209]. This effect may be related to the ability of probiotic bacteria to multiply rapidly under favorable conditions in the gut of a healthy organism [210].

Encapsulated EOs exert a beneficial influence on the biochemical parameters of animal blood and growth parameters. In particular, the feed conversion ratio (FCR), which is a principal indicator of feed efficiency, is markedly enhanced. FCR is an indicator of how effectively farm animals transform feed into the desired outcomes [211]. The selective action of EOs against probiotic bacteria may also be attributed to an enhancement in blood parameters. Hosseini and Meimandipour demonstrated a notable elevation in total protein (Tp) and globulin indices, which are contingent upon protein metabolism, nutritional conditions, and the growth of the animal [209]. These indices serve as indicators of productivity and physiological status [212].

The growth of probiotic bacteria depends on the immune status of the organism, with favorable conditions for growth directly correlating with the immune status of the organism. For example, a significant increase in the concentration of immunoglobulins Y and M has been demonstrated in studies utilizing chitosan-based particles loaded with mint, thyme, and cinnamon EOs [207]. An enhanced immune response may result in favorable alterations to the small intestinal mucosa, a region where nutrient absorption occurs. The author ascribes this effect to the influence of the chitosan matrix: the reactive functional groups (amino acids and hydroxyl groups) present in chitosan possess immunostimulatory properties and may demonstrate a synergistic effect with respect to the functions of major immune organs.

The small intestine, including the height of the villi (VH) and the depth of the crypts (CD), plays a pivotal role in the absorption of nutrients. This, in turn, has a significant impact on animal growth. The use of thymol and carvacrol, combined with various organic acids in a protein–alginate matrix, has been shown to significantly increase VH while simultaneously decreasing CD, which is both beneficial and advantageous [208,213].

In summary, the observed improvements in gut microbiota composition, biochemical markers, and intestinal morphology support the use of encapsulated EOs to enhance animal health, feed efficiency, and overall growth performance.

### 7.2. Effects on Ruminant Performance

Unlike studies in monogastric animals, research in ruminants primarily focused on antibacterial properties, metabolic modulation, and the mitigation of methane emissions, a key environmental concern (Table 7) [214].

Reducing methane emissions from agricultural ruminants is crucial due to methane’s significant impact as a greenhouse gas, possessing a global warming potential that is 28 times greater than that of carbon dioxide. Ruminants in agriculture contribute approximately 81% of greenhouse gas emissions, with about 90% of this attributed to bacterial methanogenesis [215]. Methane is produced by a group of Archaea known as methanogens, which have symbiotic relationships with various microorganisms, particularly protozoa [216]. Consequently, the methanogenesis process in these animals can be influenced by inhibiting the essential activities of methanogens or their symbiotic partners.

Soltan and colleagues showed that a combination of cinnamaldehyde, eugenol, carvacrol, and capsicum oleoresin within a lipid matrix, administered at a dosage of 400 mg/kg, significantly decreased protozoa populations in lambs [217]. Importantly, the suppression of methanogenic microorganisms is associated with lower gas emissions. Conversely, applying the same blend of encapsulated phytobiotics at a dosage of 75 g/d in beef steers led to an increase in intestinal methane emissions. The authors propose that this outcome may stem from the non-specific antibacterial properties of the encapsulated EOs, which could inadvertently enhance methanogenesis [218].

Tomkins and coauthors demonstrated that lower doses of an encapsulated EOs blend (1–2 g/d) lacked impact on the amount of gas emission [219]. The extant literature on EOs’ effect on the methanogenesis in ruminants is ambiguous. For instance, a reduction in intestinal methane emissions was observed following the administration of free EOs and their mixture in buffalo [220], whereas other studies lacked an effect on methane emissions in beef [221].

EOs can transiently reduce ruminal methane production by modulating microbiota, but adaptive microbial responses may limit their long-term efficacy [222,223]. Various EOs—including clove, eucalyptus, garlic, origanum, and peppermint oils—demonstrate significant in vitro methane inhibition by suppressing methanogenic archaea and altering bacterial populations. However, microbial adaptation over time can diminish these antimethanogenic effects [222].

Notably, EOs show differential impacts on rumen microbes and fermentation: origanum oil yields the greatest methane reduction but impairs feed digestion, whereas garlic oil lowers methane without adversely affecting digestibility. Although EOs disrupt key microbial groups linked to methanogenesis and digestion, these shifts may promote microbial resilience, curbing sustained methane mitigation [224].

In vivo evidence often reveals transient or absent methane suppression with sole EO application, suggesting limited durability due to microbiome adaptation [225]. Strategies employing optimized EOs blends at controlled dosages show potential to enhance and prolong methane reduction by potentially overcoming microbial adaptation. Thus, the rumen microbiome’s plasticity poses a significant challenge for sustained enteric methane mitigation via EOs, underscoring the need for refined combination approaches to maintain efficacy over time. Therefore, further research is required to elucidate the impact of EO dose and composition on the inhibition of methanogenic microorganisms, as well as the underlying mechanisms of action on methanogenesis.

The review of studies investigating alterations in ruminant growth parameters indicated that encapsulated EOs lacked effect on the metrics related to total nutrient intake and assimilation efficiency: dry matter intake (DMI), body weight (BW), or average daily weight gain (ADG) (Table 7).

Soltan and colleagues highlight the significance of selecting the optimal EOs dose for ruminant nutrition [217]. The administration of high doses (exceeding 400 mg/kg) of EOs resulted in a reduction in feed intake and apparent digestibility. Encapsulation of EOs can offset these undesirable effects, providing slow EOs release and preventing the rapid accumulation of excessive concentrations in the body.

**Table 7:** Effect of encapsulated EOs in livestock animals.

Wall and Core Materials (Manufacturing Company)	Levels	Animals	Effects	References
Poultry				
Sodium alginate and whey protein isolate	250 or 650 μg/g	Broilers	No effect on *Lactobacillus* spp.	[204]
Carvacrol			Non-significant reduction in *C. perfringens*	
Chitosan	60 mg/kg	Broilers	Significant increase in *Lactobacillus* spp.	[209]
Thymol			Significant reduction in *E. coli*	
Wall material n/a * (AviPlus^®^ P, Vetagro S.p.A., Reggio Emilia, Italy)	0.5%	Broilers	No effect on the *Listeria* spp., *Campylobacter* spp., and *Clostridium* spp. counts in meat	[226]
Citric acid (25.0%), sorbic acid (16.7%), thymol (1.7%), and vanillin (1.0%)				
(AviPlus^®^ P, Vetagro S.p.A., Italy)				
Sodium alginate and whey protein isolate	500 mg/kg	Broilers	Significant increase in *Lactobacillus* spp. and *Coprococcus* spp. counts.	[208]
Thymol (4%), carvacrol (4%), hexanoic acid (0.5%), benzoic acid (3.5%), and butyric acid (0.5%)			Significant reduction in *C. perfringens* counts, Bacteroidetes, Rickenellaceae.	
Wall material n/a *	0.30 g/kg	Broilers	Significant reduction in *E. coli* counts.	[227]
Sorbic acid (200 g/kg), fumaric acid (200 g/kg), and thymol (100 g/kg)				
Soy protein isolates and soluble polysaccharides	250 and 650 μg/g	Broilers	No effect on the *Lactobacillus* spp.	[228]
Citral EO			Significant reduction in *C. perfringens*	
Chitosan	100 and 200 mg/kg	Ross 308 broiler chicks	Significant increase in *Lactobacillus* spp.	[229]
Garlic EO				
Chitosan	0.025%, 0.04% and 0.055%	Ross 308 broiler chicks	Significant increase in *Lactobacillus* spp.	[207]
Mint, thyme, and cinnamon EOs			Significant reduction in *E. coli*	
Whey protein concentrate, maltodextrin, and modified starch.	0.5, 1, and 2 kg/t	Ross 308 male broiler chickens	Significant increase in *Lactobacillus* spp.	[230]
Thyme, savoury, peppermint, and black pepper EOs.			Significant reduction in *C. perfringens*	
Wall material n/a *	0.30 g/kg	Cobb 500 chicks	Non-significant increase in *Lactobacillus agilis*	[231]
Combination of organic acids and Eos (Jefo Nutrition Inc., Saint-Hyacinthe, QC, Canada)				
Wall material n/a *	150, 300, and 450 mg/kg	Hences	Significant increase in *Bifidobacterium* spp.	[232]
Sorbic acid (200 g/kg), fumaric acid (200 g/kg), and thymol (100 g/kg)				
Pigs				
Wall material n/a * Citric acid (25%), sorbic acid (16.7%), thymol (1.7%), and vanillin (1%) (Aviplus-S^®^, Vetagro S.p.A., Italy)	0.2%	Weaned pigs	No effect on *Lactobacillus* spp. and *E. coli* counts	[233]
Triglycerides from hydrogenated vegetable oil Fumaric acid, citric acid, malic acid, sorbic acid, thymol, vanillin, and eugenol (Jefo Nutrition Inc., Canada)	1 and 2 g/kg	Weaned pigs	Significant increase in *Lactobacillus* and *Bacilli*	[234]
Wall material n/a * Formic acid, citric acid, citrus, cinnamon, oregano, thyme, and capsicum EOs (FormaXOL™, Kemin Industries, Des Moines, IA, USA)	4 kg/t	Finishing pigs	Significant reduction in *Salmonella* spp.	[235]
Ruminants				
Wall material n/a * Cinnamaldehyde and garlic EO (Cargill, Minnetonka, MN, USA)	300 mg/d	Drylot beef cattle	No effect on ADG, BW No effect on fecal egg count No effect on glucose and urea nitrogen Non-significant reduction of horn fly population	[236]
Wall material n/a * Carvacrol, cinnamaldehyde, eugenol, and capsaicin from capsicum oleoresin (Activo^®^ Premium, GRASP Ind. e Com. Ltd.a, Curitiba, Brazil)	75 g/d	Steers	No effect on FBW, ADG, gain to feed ratio, DMI. No effect on blood partial pressure of carbon dioxide (pCO_2_) and oxygen (pO_2_), total concentration of CO_2_ (tCO_2_), saturation of O_2_ (SatO_2_) and CO_2_ (SatCO_2_), bicarbonate (HCO_3_^−^), total Hb, base excess (BE), pH, and packed cell volume (PCV) Non-significant increase in CH_4_ emission.	[218]
Organic carrier Thymol, eugenol, vanillin, limonene, and guaiacol (CRINA^®^ Ruminants, DSM Nutritional Products Ltd., Kaiseraugst, Switzerland)	1 or 2 g/d	Rumen fistulated Brahman (BBos. indicus) steers	No effect on VFA concentration No effect on CH_4_ emission	[219]
Wall material n/a * Carvacrol, cinnamaldehyde, eugenol, and capsaicin from capsicum oleoresin (Activo^®^ Premium, GRASP Ind. e Com. Ltd.a, Brazil)	150 mg/kg	Steers	No effect on FBW, ADG, DMI. No effect on carcass characteristics: the HCW, DP, marbling score, back fat depth, percentage of carcasses classified as low choice or greater, as well as incidence of liver abscess. Non-significant increase in YG, back fat depth.	[237]
Wall material n/a * Eugenol, thymol, and vanillin	4 g/animal/day	Nellore heifers	No effect on the meat chemical composition or the muscle fatty acid profile. Increase in sarcomere length, soluble collagen content. Reduction in type III collagen.	[238]
Wall material n/a * Eugenol, thymol, and vanillin	4 g/animal/day	Feedlot-finished heifers	No effect on pH of meat, fat thickness, intramuscular fat, or meat tenderness. Significant increase in antioxidant activity in meat. Significant reduction in lipid peroxidation in meat and meat color degradation.	[239]
Lipid matrix (80%) Anethole (10%) and carvone (10%)	50 mg/kg	Lambs	No effect on BWG of animals infected with *H. contortus* compared to uninfected, *H. contortus* counts (at dose 20 mg/kg). Significant reduction in FEC.	[240]
Fat matrix Cinnamaldehyde, eugenol, carvacrol, and capsicum oleoresin	200 and 400 mg/kg	Sheep	No effect on FI, DM. Significant reduction in CH_4_ emissions and protozoa counts.	[217]

* n/a—not available.

The robustness in livestock species has garnered increasing attention in response to climate change and agroecological transformations, all of which subject animals to diverse environmental stressors. Generally, key aspects that determine robustness in animal in vivo studies are biological variability related to species, strain, and health status; environmental factors, including housing, diet, and handling; and critical elements of experimental design like randomization, replication, and use of controls [241]. Importantly, references in Table 7 provide detailed specifications of experimental conditions, species, strain, and health status, and incorporate appropriate controls, which strengthen the reliability and robustness of their findings.

### 7.3. Effects on Gut Microbiota and Host Immunity

Encapsulated EOs, through advanced delivery systems, exert targeted effects on gut microbiota, mucosal immunity, epithelial integrity, and oxidative stress responses.

Encapsulated EOs have been demonstrated to enhance serum concentrations of immunoglobulin G (IgG) and immunoglobulin A (IgA), both of which are integral to mucosal immunity. These compounds also modulate the immune response by upregulating anti-inflammatory cytokines such as interleukin-10 (IL-10) and downregulating pro-inflammatory cytokines, including tumor necrosis factor-alpha (TNF-α) and interleukin-1 beta (IL-1β), thereby exerting immunomodulatory effects. Mechanistically, encapsulated EOs activate the nuclear factor erythroid 2-related factor 2 (Nrf2) signaling pathway, which is pivotal for cellular antioxidant defense, while concurrently inhibiting the Toll-like receptor 4 (TLR4)/nuclear factor kappa B (NF-κB) pathway, leading to attenuation of mucosal inflammatory responses and maintenance of tissue homeostasis. Furthermore, these encapsulated compounds enhance both the abundance and secretory function of goblet cells, which are responsible for mucin production, thus strengthening the intestinal mucus barrier and reducing susceptibility to pathogenic invasion [242,243].

Encapsulated EOs significantly upregulate the expression of key tight junction proteins, including zonula occludens-1, occludin, and claudin-1, in intestinal epithelial cells. This modulation enhances the intestinal barrier function, as evidenced by decreased gut permeability and reduced serum biomarkers such as D-lactic acid and diamine oxidase activity. The reinforcement of tight junction assembly by encapsulated EOs limits the translocation of toxins and pathogenic bacteria, thereby preserving gut integrity, which is particularly crucial during physiological stressors like weaning or infectious challenges. Furthermore, the materials utilized for microencapsulation, such as chitosan and modified starch, may exhibit prebiotic properties that further support barrier function by promoting the growth of beneficial microbiota and stimulating short-chain fatty acid (SCFA) production [242,244,245]. This integrated mechanism highlights the potential of encapsulated EOs both as modulators of epithelial barrier integrity and as functional agents in maintaining intestinal health under stress conditions relevant to antimicrobial stewardship and gut-related pathologies.

In pigs and broiler chickens, encapsulated EOs enhance the activity of key intestinal antioxidant enzymes, including superoxide dismutase and glutathione peroxidase. This enzymatic upregulation is associated with a reduction in MDA concentrations, a recognized biomarker of lipid peroxidation and oxidative stress. Through modulation of inflammatory signaling pathways and activation of the Nrf2 pathway, encapsulated EOs mitigate reactive oxygen species (ROS)-induced damage to intestinal epithelial cells. Furthermore, the observed upregulation of antioxidant defense-related gene expression coupled with the downregulation of pro-inflammatory mediator transcripts at the mRNA level contributes to the maintenance of redox homeostasis within the gut environment [208,242].

Encapsulated EOs exhibit selective antimicrobial activity by inhibiting pathogenic bacteria while preserving or promoting beneficial microbiota, including Lactobacillus species. This selective modulation contributes to the maintenance of microbial diversity and metabolic functionality within the gut ecosystem. Furthermore, evidence indicates an enhancement in the production of SCFAs, such as butyrate and lactate, which support colonocyte health and reinforce epithelial barrier integrity [245,246,247].

Thus, encapsulated EOs exert various effects on the gut ecosystem. Studies show their capability to modulate the immune response, reinforce epithelial barriers, counter oxidative stress, and sculpt microbial communities.

## 8. Conclusions and Future Perspectives

Given the limited use of feed antibiotics and the rising resistance of pathogens, EOs could play a significant role in animal nutrition. While the antibacterial properties of EOs against pathogenic bacteria have been studied extensively, their impact on probiotic bacteria remains unclear.

EOs encapsulation in natural matrices is a sensible approach that enhances EOs’ stability. Encapsulation also protects EOs from rapid degradation in the harsh conditions of the gastrointestinal tract, allowing for a gradual release. Such controlled release minimizes the risk of a burst EO release within the body, which can lead to decreased feed intake and apparent digestibility, ultimately affecting animal growth performance [217].

Spray drying and complex coacervation are the most reproducible and scalable techniques for obtaining particles loaded with EOs. These methods are suitable for encapsulation into multicomponent matrices. Utilizing multicomponent matrices is a modern trend in EOs encapsulation for animal nutrition, enabling the optimization of both economic and physicochemical characteristics of the particles.

In monogastric animals, various EOs encapsulated in a chitosan matrix or a polysaccharide–protein matrix demonstrated a notable increase in the growth of probiotic bacteria. Moreover, some studies reported a significant reduction in the growth of pathogenic microflora.

EOs’ antibacterial properties against methane-producing bacteria in ruminants may help lower methane emissions into the atmosphere, thereby decreasing greenhouse gas levels. But, research regarding the appropriate dosages and mechanisms by which encapsulated EOs influence methane emissions in ruminants is lacking in the literature. This issue remains unclear and requires further investigation.

Despite the numerous positive effects and the extensively researched mechanisms behind EOs’ antimicrobial properties, several aspects remain ambiguous. For instance, the reasons why EOs positively influence probiotic microorganisms in vivo while simultaneously inhibiting pathogenic growth are not fully understood. Additionally, evaluating the effectiveness of encapsulated EOs is challenging due to the limited studies conducted on both monogastric and ruminant animals. Furthermore, the existing literature often fails to provide a complete composition of the matrix, complicating the interpretation of findings.

Thus, further studies are needed in all types of farm animals to identify appropriate dosages, particle core compositions, and matrix formulations for the effective application of encapsulated EOs as an alternative to feed antibiotics.

Also, current data on the co-encapsulation of multiple essential oils for synergistic effects are limited and poorly characterized, highlighting the need for more comprehensive research. The impact of encapsulation matrices on bioactive interactions remains unclear, and compatibility issues and formulation challenges are underexplored. These gaps present important opportunities for future investigation to advance understanding in this field.

## Data Availability

The original contributions presented in this study are included in this article. Further inquiries can be directed to the corresponding authors.

## Abbreviations

The following abbreviations are used in this manuscript:

ADG	Average daily weight gain
AGPs	Antibiotic growth promoters
ATP	Adenosine triphosphate
BW	Body weight
CD	Depth of the crypts
DMI	Dry matter intake
DNA	Deoxyribonucleic acid
DSC	Differential scanning calorimetry
DTG	Differential thermogravimetric analysis
EE	Encapsulation efficacy
EOs	Essential oils
ESD	Emulsification–solvent diffusion
ESE	Emulsification–solvent evaporation
FCR	Feed conversion ratio
FDA	Food and Drug Administration
GI	Gastrointestinal
GRAS	Generally recognized as safe
IgA	Immunoglobulin A
IgG	Immunoglobulin G
IL-10	Interleukin-10
IL-1β	Interleukin-1 beta
MDA	Malondialdehyde
NF-κB	Nuclear factor kappa B
NLC	Nanostructured lipid carriers
Nrf2	Nuclear factor erythroid 2-related factor 2
NSP	Non-starch polysaccharides
OSA	Octenylsuccinate
PBS	Phosphate-buffered saline
PDI	Polydispersity index
RNA	Ribonucleic acid
ROS	Reactive oxygen species
SCFA	Short-chain fatty acid
SGF	Simulated gastric fluid
SIF	Simulated intestinal fluid
SLN	Solid lipid nanoparticles
TG	Thermogravimetry
TGA	Thermogravimetric analysis
TLR4	Toll-like receptor 4
TNF-α	Tumor necrosis factor-alpha
Tp	Total protein
TPP	Triphenyl phosphate
VH	Height of the villi
VOCs	Volatile organic compounds
